# Sleep extension is a feasible lifestyle intervention in free-living adults who are habitually short sleepers: a potential strategy for decreasing intake of free sugars? A randomized controlled pilot study

**DOI:** 10.1093/ajcn/nqx030

**Published:** 2018-01-10

**Authors:** Haya K Al Khatib, Wendy L Hall, Alice Creedon, Emily Ooi, Tala Masri, Laura McGowan, Scott V Harding, Julia Darzi, Gerda K Pot

**Affiliations:** 1 Diabetes and Nutritional Sciences Division, King's College London, London, United Kingdom; 2 Centre for Public Health, School of Medicine, Dentistry, and Biomedical Sciences, Queens University Belfast, Belfast, United Kingdom; 3 Faculty of Earth and Life Sciences, Section of Health, Department of Biochemistry, Memorial University of Newfoundland, St. John's, Canada; 4 Department of Health and Life, Vrije Universiteit Amsterdam, Amsterdam, Netherlands

**Keywords:** sleep extension, energy balance, energy intake, diet, randomized controlled trial, RCT

## Abstract

**Background:**

Evidence suggests that short sleep duration may be a newly identified modifiable risk factor for obesity, yet there is a paucity of studies to investigate this.

**Objective:**

We assessed the feasibility of a personalized sleep extension protocol in adults aged 18–64 y who are habitually short sleepers (5 to <7 h), with sleep primarily measured by wrist actigraphy. In addition, we collected pilot data to assess the effects of extended sleep on dietary intake and quality measured by 7-d food diaries, resting and total energy expenditure, physical activity, and markers of cardiometabolic health.

**Design:**

Forty-two normal-weight healthy participants who were habitually short sleepers completed this free-living, 4-wk, parallel-design randomized controlled trial. The sleep extension group (*n* = 21) received a behavioral consultation session targeting sleep hygiene. The control group (*n* = 21) maintained habitual short sleep.

**Results:**

Rates of participation, attrition, and compliance were 100%, 6.5%, and 85.7%, respectively. The sleep extension group significantly increased time in bed [0:55 hours:minutes (h:mm); 95% CI: 0:37, 1:12 h:mm], sleep period (0:47 h:mm; 95% CI: 0:29, 1:05 h:mm), and sleep duration (0:21 h:mm; 95% CI: 0:06, 0:36 h:mm) compared with the control group. Sleep extension led to reduced intake of free sugars (–9.6 g; 95% CI: –16.0, –3.1 g) compared with control (0.7 g; 95% CI: –5.7, 7.2 g) (*P* = 0.042). A sensitivity analysis in plausible reporters showed that the sleep extension group reduced intakes of fat (percentage), carbohydrates (grams), and free sugars (grams) in comparison to the control group. There were no significant differences between groups in markers of energy balance or cardiometabolic health.

**Conclusions:**

We showed the feasibility of extending sleep in adult short sleepers. Sleep extension led to reduced free sugar intakes and may be a viable strategy to facilitate limiting excessive consumption of free sugars in an obesity-promoting environment. This trial was registered at www.clinicaltrials.gov as NCT02787577.

## INTRODUCTION

Sleep is increasingly recognized as a potential modifiable risk factor that may be involved in the complex etiology of obesity and cardiometabolic diseases (1) and is becoming an area of increasing public health concern (2). Observational studies showed links between short sleep duration or poor sleep quality with weight gain (3–5) and its associated cardiometabolic complications (6–11). Today, ∼37% of US adults report sleeping ≤6 h, and less than two-thirds are achieving the recommended 7–9 h/night for optimal physical and mental well-being (12, 13).

Experimental evidence investigating the effects of sleep deprivation on insulin resistance (14–16) and glucose (17–19) and appetite hormone (20, 21) dysregulation has indicated that poor sleep is potentially detrimental to overall health. In our recent systematic review and meta-analysis of intervention studies, we found that partial sleep deprivation caused a 385-kcal (95% CI: 252-, 517-kcal) increase in energy intake in comparison to the control habitual sleep condition, with no compensatory effects on energy expenditure (22). If sustained, the net positive energy balance due to sleep deprivation may manifest in weight gain, which was previously shown after 5 nights of restricting sleep to 4 h (23). The majority of sleep deprivation studies to date are highly restrictive (<5 h/night) and conducted acutely (<2 wk) in controlled laboratory settings. Studies in ecologically valid conditions are needed to distinguish whether laboratory evidence applies to free-living conditions. Moreover, the poor dietary habits that are characteristic of short sleepers reported in observational studies have been extensively reviewed (24), yet the relation has yet to be investigated in randomized controlled trials (RCTs).

Turning the relation the other way around, evidence from a longitudinal study in adults has shown that spontaneously shifting sleep duration from a short to a healthier length has been associated with attenuated fat mass gain (25). Yet, few longer-term (≥1 mo) RCTs have assessed the effects of sleep extension (SE) in free-living adult short sleepers, and these were mainly conducted in prehypertensive individuals with the aim of improving blood pressure (26, 27).

The aim of this research was to assess the feasibility of SE by using a behavioral change approach targeting sleep hygiene under free-living conditions in healthy adults who are habitually short sleepers. Primary outcomes were objective measures of compliance to the sleep hygiene intervention in order to determine the feasibility of the SE intervention. Other feasibility measures included rates of subject participation and attrition. If it was shown to be feasible to extend sleep duration in a free-living population, it was hypothesized that SE would lead to changes in secondary outcome measures that are conducive to maintenance of weight and cardiometabolic health. To test this hypothesis, the secondary aim of the study was to conduct a pilot investigation on the effects of SE on dietary intake and indicators of energy balance. Exploratory analyses of markers of cardiometabolic risk, appetite hormones, and heart rate variability were included to provide preliminary data for use in planning sample sizes in future, larger SE intervention studies.

## METHODS

### Experimental design

The Sleep Lengthening and Metabolic health, Body composition, Energy balance and cardiovascular Risk (SLuMBER) Study is an open-label, 4-wk parallel-design RCT investigating the feasibility of SE in habitually short sleepers and its effects on dietary intake, energy balance, and cardiometabolic health indicators in comparison to a control, habitual short sleep condition. The protocol was approved by the King's College London (KCL) Research Ethics Committee (HR-15/16-2172) and conducted in line with the Declaration of Helsinki. The trial is registered at www.clinicaltrials.gov (NCT02787577).

### Participants

Healthy men and women aged 18–64 y and with a BMI (in kg/m^2^) of 18.5 to <30 were recruited by using internal circular e-mails among KCL staff and students, as well as social media advertisements and recruitment posters that were publicly available. Respondents to advertisements were initially screened by telephone or e-mail questionnaires. Inclusion criteria as assessed by the screening questionnaire specified healthy men and women with a BMI of 18.5 to <30 who were habitual short sleepers, defined as a self-reported average sleep duration of 5 to <7 h/night on weeknights, which was confirmed with the use of baseline actigraphy measurements. Exclusion criteria included the following: diagnosed medical conditions such as cardiovascular disease, type 1 or 2 diabetes, cancer, chronic liver or renal disease, inflammatory bowel disease, and thyroid conditions. Participants were also excluded if they reported a weight change >3 kg in the previous 2 mo, substance or alcohol abuse (>28 units/wk for men; >21 units/wk for women), use of antidepressants or chronic use of sleep medication, smoking, shift work, habitual napping, an obligation to wake up at night to care for others, and an inability to adhere to a sleep intervention due to time commitments. Participants with an extreme chronotype according to the Morningness-Eveningness questionnaire (28) (defined as a score of ≥70 or ≤30) were excluded from the study. Those at a high risk of low mood or any sleep-related disorders according to the Center of Epidemiologic Studies–Depression scale (CES-D) (29), the Epworth Sleepiness Scale (ESS) (30), the Insomnia Severity Index (ISI) (31), or the Berlin Questionnaire for sleep apnea (32) were excluded from the study.

Eligible participants were then invited for a clinical screening visit where their BMI was evaluated, and a fasted blood sample was taken to analyze liver function, glucose, lipids, and hematology at an accredited clinical biochemistry laboratory (ViaPath, King's College Hospital) on the same day. Written informed consent was obtained before any measures were taken. Eligible participants were enrolled in the study. All of the visits were scheduled such that no participants had trans-meridian travel within 4 wk of commencing the study. Participants received travel reimbursements (≤£10/visit) and were compensated £75 upon completion of the study.

### Clinical visits and study measures, and randomization

**FIGURE 1:**
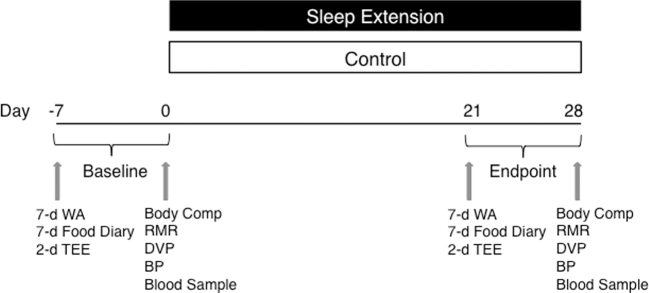
Illustration showing the 4-wk sleep extension parallel, randomized-controlled research design. Baseline and endpoint measures were collected before randomization and within the last week of the trial, respectively. Body Comp, body composition; BP, blood pressure; DVP, digital volume pulse; RMR, resting metabolic rate; TEE, total energy expenditure; WA, wrist actigraphy.

All of the clinical visits were conducted in the Metabolic Research Unit at KCL, Waterloo Campus. All outcomes of the study were assessed at baseline (before randomization) and at endpoint during the fourth week of the study, as shown in **Figure 1**.

Participants attended 2 clinical study visits at baseline and again at the endpoint. At the first visit, participants were fitted with a wrist actigraph (MotionWatch8; CamNTech Ltd.) to wear on their nondominant wrist for 7 d (7 nights). Participants were asked to log their sleep and wake times in 7-d sleep diaries and to complete a 7-d food diary. To measure total energy expenditure (TEE), participants were fitted with an Actiheart (CamNTech Ltd.), a small, light-weight, chest-worn unit that combines a heart rate monitor and accelerometer to wear for 48 h. Participants then completed the Dutch Eating Behavior Questionnaire (33) to assess eating behavior, in addition to other questionnaires outlined below.

The second clinical visit was scheduled a minimum of 8 d after the first visit. Participants arrived after a 12-h overnight fast and were instructed to refrain from intense physical exercise for the previous 24 h. Baseline wrist actigraphy measures were appraised to confirm that participants were short sleepers. Height was measured to the nearest 0.01 m. Body composition was measured with the use of a segmental bioelectric impedance analyzer (Tanita BC-418; Tanita Corporation of America, Inc.). Three waist circumference measurements to the nearest 0.1 cm at the midpoint between the lowest rib and iliac crest were taken to obtain an average. Participants were then instructed to lie on a bed in a semirecumbent position and remain relaxed but awake for 20 min before resting metabolic rate (RMR) assessment. RMR was measured by indirect calorimetry with the use of the FitMate (CosMed), a previously validated (34, 35) metabolic analyzer that measures oxygen consumption under a hood to estimate energy expenditure. Pulse Trace (Micro Medical Ltd.), a portable digital volume pulse machine that uses photoplethysmography in a finger-clip probe attached to the participant’s index finger, assessed vascular function over a 15-s period. Recordings were taken in triplicate, and an average was calculated. Blood pressure measurements were then taken in triplicate with the use of an A&D Medical UA-767 Plus auto upper arm blood pressure monitor, in accordance with the British Hypertension Society Guidelines (36). A fasting blood sample was taken and processed on the same day; aliquots were then stored at −80°C for later analysis.

Upon completion of all measurements, participants were randomly assigned (1:1), with stratification to minimize the difference between groups for age, sex, BMI, and ethnicity (performed by MinimPy 0.3 randomization computer program accessed at https://sourceforge.net/projects/minimpy/) by the researchers, and informed whether they were enrolled to the SE group or to a control group. The SE group received an SE intervention, as detailed below. The control group was asked to resume their lifestyle as usual, and informed that they would receive the intervention session upon completion of the study.

### SE intervention

The SE intervention entailed a personalized sleep consultation session (∼45 min), with the goal of extending time in bed (TIB) by 1–1.5 h/night. The intervention was designed in consultation with a health psychologist to focus on improving participants’ sleep hygiene with the use of evidence-based behavior change techniques by consulting the ``Coventry, Aberdeen & London - Refined'' (CALO-RE) taxonomy (37). The personalized consultation session was delivered with the use of a script to standardize the sessions. First, the importance of sleep, current sleep recommendations (7–9 h), and the concept of sleep hygiene were explained to the participant. Participants were informed that the success of the intervention was dependent on the consultant and the participant working together to design a strategy that could most feasibly be implemented in the participant's current lifestyle. Next, the participant was provided with a list of sleep hygiene behaviors that was formulated with guidance from publicly accessible resources, such as the Harvard Division of Sleep Medicine's Twelve Simple Tips to Improve Your Sleep (38) and the National Sleep Foundation (39), American Sleep Association (40), and the UK National Health Service (41) websites. With regard to sleep hygiene practices that are of relevance to diet, participants were informed that excessive caffeine intake late in the day, as well as going to bed too full or too hungry, could disrupt their ability to go to sleep. The consultant reviewed all of the sleep hygiene behaviors with the participant and encouraged the participant to identify any that were relevant to his or her lifestyle. The participants were then supported with a selection of a minimum of 4 sleep hygiene behaviors they felt were most applicable and easily implemented in their lifestyle, and these were documented in a personalized sleep diary for their own reference. Participants identified barriers (37) to achieving their selected behaviors, and were assisted to create implementation intentions (37) that were also noted in the personalized sleep diary. Participants were prescribed a recommended bedtime to increase their TIB, and this was outlined as a “behavioral contract” (37), which lists the agreed-on selected behaviors and bedtimes. Participants completed the sleep diary by noting sleep and wake times and indicated whether they achieved the selected sleep hygiene behaviors on the day for self-monitoring (37) purposes throughout the duration of their intervention period.

### Sleep assessment

Sleep was assessed objectively by wrist actigraphy, which has been validated against the gold standard for sleep assessment, polysomnography (42). The MotionWatch8 was programmed to record motions every 30-s epoch and set at a threshold of 20 s to distinguish sleep from waking, because this high-sensitivity setting yielded the clearest agreement with a polysomnography validation study (43, 44). While wearing the wrist actigraph, participants were asked to push an “event marker” button on the device to indicate their intention to go to sleep and again when they woke up in the morning with no intent to return to sleep. Recordings were downloaded to the MotionWare Software (CamNTech) for analysis. Participants’ sleep diaries and event marks were used to define the TIB period on the software (from “lights out” to “got up”). Once this sleep region was selected, the software automatically calculated the times at which participants “fell asleep” and “woke up” to define “sleep period.” “Sleep duration” was automatically defined as the total time spent asleep according to epoch-by-epoch sleep/wake categorization within the sleep period, therefore excluding time awake. “Sleep latency” was the time between “lights out” and “fell asleep.” “Sleep efficiency” was calculated as sleep duration expressed as a percentage of TIB. “Sleep duration (%)” was sleep duration expressed as a percentage of sleep period. Sleep Fragmentation Index (SFI) denotes the degree of sleep fragmentation, where a higher SFI indicates poorer sleep quality. A 7-d average was calculated for each sleep variable.

Subjective measures of sleep quality were also assessed with the use of the Pittsburgh Sleep Quality Index (45). The Sleep Hygiene Index (46) was used to verify adherence to behaviors of sleep hygiene.

### Dietary assessment

To assess dietary intake, we used 7-d estimated food diaries that resembled the UK National Diet and Nutrition Survey Rolling Program diaries (47), with predefined time slots for food intake. Estimated food diaries were selected, because this method was deemed to show the greatest magnitude of agreement of numerous nutrients against the gold standard of self-reported dietary intake, the weighed food diary (Spearman rank = 0.35–0.83) in comparison to food-frequency questionnaire (Spearman rank = 0.39–0.57) and 24-h recall (Spearman rank = 0.21–0.63) (48). Moreover, intakes reported in the estimated diaries showed good correlation with 24-h urinary excretion of nitrogen and potassium (Spearman rank = 0.65 and 0.66, respectively) (48) and have been shown to provide better estimates than food-frequency questionnaires (49). Estimated rather than weighed food diaries were used due to their utility in the free-living setting, as well as to minimize participant burden. Participants were instructed to provide as much detail as possible about all food, drinks, and supplements consumed, and encouraged to attach packaging. Portion sizes were reported using household measures or in grams, as well as estimated with the use of visual guide portion-size photographs included in the diary. Mean daily intakes were analyzed with the use of Nutritics (Nutritics Professional Diet Analysis, version 3.74; Nutritics Ltd.), which incorporates McCance and Widdowson's 6th edition of *The Composition of Foods*. Seven-day averages were calculated for all nutrients and were assessed in grams as well as percentages of total energy, where appropriate. Dietary misreporters were identified with the use of previously outlined methods by McCrory et al. (50) for sensitivity analysis on plausible reporters with the use of 2-SD cutoffs.

### Diet quality and UK dietary guidelines adherence assessment

Because there is currently no diet quality index available in the United Kingdom, we used the Eating Choices Index (51) and formulated a composite score to assess adherence to the UK dietary guidelines (52, 53) with the use of the 7-d food diaries. The following 6 UK dietary guidelines were used: 5 portions of fruit and vegetables, ≥30 g fiber, <6 g salt, <11% of energy from saturated fat, <5% of energy from free sugars, and ≥2 portions of fish/wk (≥1 of which is oily). Each of the 7 d of the food diary was scored such that participants received a score of “1” if they met the daily requirement for the given category and “0” if not. However, because guidelines for fish intake are weekly and not daily, a score of 3.5 was given if ≥2 portions of fish were consumed and 3.5 was given if ≥1 portion of fish was oily in order to avoid over- or underrepresenting this category. The maximum score for each category was 7, and summed across the 7 d; the overall maximum adherence score was 42, where a higher score indicates a healthier diet that complies more closely with the current guidelines.

### TEE and physical activity assessment

The Actiheart has been previously validated against doubly labeled water (54, 55), a gold standard for the assessment of TEE in free-living conditions. The monitor was fitted to the chest with the use of 2 electrocardiogram electrodes (SP-50, 50 mm round; Pulse Medical) for attachment. Before placement of electrodes, the skin was shaved of any chest hair and wiped with the use of alcohol wipes to clean, and an abrasive pad (Unilect) was used to remove the top layer of skin cells. A signal test was recorded to confirm the quality of the signal. In addition, a step-test was performed to measure participants’ heart rate recovery, allowing for calibration of the recordings to participants’ fitness level. The 48-h recordings were programmed by using the short-term inter-beat intervals application, allowing for analysis of heart rate variability parameters, a measure of autonomic function, as outlined previously (56). Data were processed and analyzed with the use of the Actiheart software (version 4.0.116; CamNTech Ltd.).

Physical activity intensity was analyzed with the use of the MotionWatch8 with the use of MotionWare software's Daytime analysis application. Seven-day average active times spent in vigorous, moderate, low, and sedentary activity that were determined by the software were extracted and calculated as a percentage of time spent awake or in “active time” as defined by the software.

### Circulating markers of cardiometabolic risk

All of the analyses were conducted on stored (−80°C) serum and plasma samples. Serum nonesterified fatty acids (RANDOX NEFA kit), serum cholesterol profile (IL Test; Instrumentation Laboratory Ltd.), plasma triglycerides (IL Test), and plasma glucose (Glucose Oxidase kit) were determined with the use of enzymatic colorimetric assays on the ILAB 650 Clinical Chemical System (Instrumentation Laboratory Ltd.). Immunoassays were conducted by using the ADVIA 2400 analyzer (Siemens Healthcare Diagnostics Ltd.) to measure concentrations of serum insulin (Siemens Healthcare Diagnostics Ltd.), serum c-peptide (Siemens Healthcare Diagnostics Ltd.), serum cortisol (Siemens Healthcare Diagnostics Ltd.), serum leptin (Quantikine ELISA kits, R&D Systems, Abingdon, UK), and plasma ghrelin (Ghrelin RIA kit; Millipore Corporation). We used the HOMA-IR (57), where the product of fasting glucose and fasting insulin is an index of insulin resistance. This was calculated as follows: HOMA-IR = [fasting glucose (mmol/L) × fasting insulin (mU/L)]/22.5.

### Statistical analyses

A sample size of *n* = 40 participants (20 participants/group) was determined to be sufficient for investigating the feasibility of the SE protocol (58, 59) and to gain SD data to inform statistical power calculations for future RCTs. Primary outcomes included measures of sleep duration (TIB, sleep period, and sleep duration). Rates of participation and attrition were also calculated as feasibility outcomes (58, 59). The intervention was to be deemed feasible if sleep duration was extended, the attrition rate was <20% (95% CI: 8%, 30%), and ≥80% (95% CI: 68%, 92%) of all recruited participants completed follow-up. All data were analyzed with the use of a modified intention-to-treat analysis, where missing data due to technical difficulties with equipment or inability to draw blood were not included in the analysis. Data were tested for normality by visual evaluation of histograms and Q-Q plots and confirmed by the Shapiro-Wilk test. Non-normally distributed data were log-transformed and retested; nonparametric analysis was applied if data still did not conform to a normal distribution. Participant characteristics were assessed with the use of an independent-samples *t* test in order to detect significant differences between groups at screening. A chi-square test was applied to assess nominal data. Sleep variables were analyzed by ANCOVA on the change from baseline, with baseline values included as a covariate.

The secondary outcomes were assessed with the use of ANCOVA on change from baseline, and covariates included were baseline measures for the given outcome, as well as the change from baseline in TIB to account for compliance. Nonparametric change from baseline data was assessed with the use of a Mann-Whitney *U* test to compare differences between groups. Within-group comparisons were assessed on baseline and endpoint data by a paired-samples *t* test for a Wilcoxon’s Signed Rank test for normal and nonparametric data, respectively. Data are expressed as means (95% CIs), medians (IQRs), or numbers [*n* (% of total)]. Statistical analysis was carried out with the use of IBM SPSS Statistics 22.0 (Statistical Product and Service Solutions; IBM Corp.). A trend was considered when *P* values were between 0.05 and <0.1. Differences were considered significant at the level of *P* < 0.05.

## RESULTS

### Participant characteristics, rates of participation, and attrition

**FIGURE 2:**
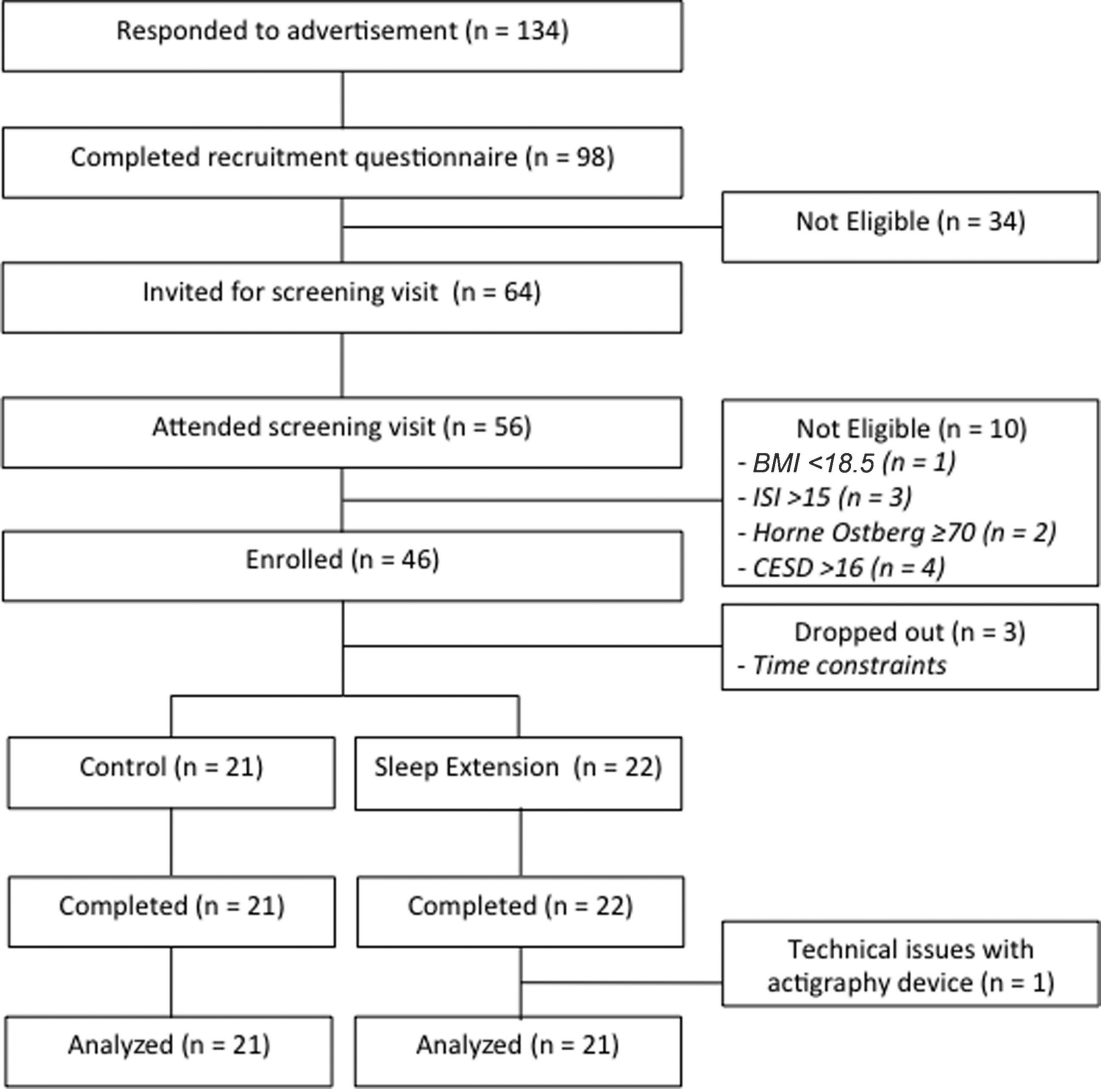
CONSORT diagram of the SLuMBER study. CESD, Center of Epidemiologic Studies–Depression scale; CONSORT, Consolidated Standards of Reporting Trials; ISI, Insomnia Severity Index; SLuMBER, Sleep Lengthening and Metabolic health, Body composition, Energy balance and cardiovascular Risk Study.

Of the 46 participants eligible for the study, 46 (100%) enrolled, and 43 completed the study, as shown in **Figure 2**. One participant was removed from all analyses due to technical difficulties with the wrist actigraphy device, and therefore the inability to adjust for compliance (change from baseline in TIB) for secondary outcomes. There was a 6.5% rate of attrition, because 3 participants dropped out from the study during baseline measurements due to time constraints before being informed of the treatment group to which they were allocated. There were no dropouts after random assignment to treatment. Baseline wrist actigraphy measures indicated the all of the enrolled participants were short sleepers, and therefore none were excluded. There were no differences in the distribution of sex, age, ethnicity, or BMI across groups, confirming that the stratified randomization was successful, as indicated in **Table 1**. BMI in both groups was within the normal healthy range (18.4–24.9). There were no significant differences at screening for fasting blood glucose or blood lipid profile between groups, and mean values were within normal ranges. There were no significant differences between groups in scores for screening questionnaires used to detect sleep disorders (Horne-Ostberg, Insomnia Severity Index, Epworth Sleepiness Scale) and risk of depressed mood (Center of Epidemiologic Studies–Depression scale). There were no markedly predominant eating behaviors in the groups or any significant differences across groups as assessed by the Dutch Eating Behavior Questionnaire.

**TABLE 1:** Participant characteristics of the SLuMBER study as assessed at screening^1^

	Control group (*n* = 21)	Sleep extension participants (*n* = 21)
Sex, *n* (%)		
Male	4.0 (9.5)	3.0 (7.1)
Female	17 (40.5)	18 (41.9)
Age,^2^ y	25.0 [22.5, 30.5]	24.0 [21.5, 29.0]
Ethnicity, *n* (%)		
White	12 (28.6)	11 (26.2)
Black	1 (2.4)	2 (4.8)
South/East Asian	4 (9.5)	5 (11.9)
Other	4 (9.5)	3 (7.1)
BMI,^3^ kg/m^2^	21.8 (20.9, 22.8)	22.5 (21.3, 23.8)
Glucose,^3,4^ mmol/L	5.0 (4.7, 5.3)	4.7 (4.6, 4.9)
TC,^3,4^ mmol/L	4.5 (4.2, 4.7)	4.4 (4.0, 4.8)
TGs,^3,4^ mmol/L	0.8 (0.6, 0.9)	0.8 (0.6, 0.9)
HDL cholesterol,^3,4^ mmol/L	1.7 (1.6, 1.9)	1.6 (1.4, 1.9)
LDL cholesterol,^3,4^ mmol/L	2.4 (2.0, 2.8)	2.4 (2.2, 2.7)
Sleep score		
Horne-Ostberg	52.6 (48.6, 56.6)	50.7 (47.5, 53.9)
ISI	7.9 (6.4, 9.3)	7.6 (6.1, 9.1)
ESS^2^	6.0 [5.0, 6.8]	6.0 [4.0, 7.0]
Depressed mood score (CES-D)	7.3 (5.5, 9.0)	5.9 (3.9, 7.8)
Eating behavior score (DEBQ)		
Restrained	2.7 (2.3, 3.2)	2.7 (2.4, 3.0)
Emotional	2.3 (1.9, 2.7)	2.4 (2.1, 2.8)
External	3.1 (2.8, 3.4)	3.3 (3.0, 3.7)

1 Data were tested with the use of chi-square test, independent-samples *t* test, or Mann-Whitney *U* test. There were no significant differences between groups (*P* values not shown). CES-D, Center of Epidemiologic Studies–Depression scale; DEBQ, Dutch Eating Behavior Questionnaire; ESS, Epworth Sleepiness Scale; ISI, Insomnia Severity Index; SLuMBER, Sleep Lengthening and Metabolic health, Body composition, Energy balance and cardiovascular Risk Study; TC, total cholesterol; TG, triglyceride.

2 Values are medians [IQRs].

3 Values are geometric means (95% CIs). Data were log-transformed before analysis.

4 Data from 2 participants are missing from analysis due to inability to draw blood (Control group: *n* = 20, sleep extension participants: *n* = 20)

### Intervention compliance

#### Sleep duration

The differences in outcomes of 7-d sleep duration and quality between the SE and control groups as a result of the SE intervention are outlined in **Table 2**. At endpoint, the change from baseline was significantly higher in the SE group for TIB, sleep period, and sleep duration in comparison to the control group. In the SE group, 86% of participants increased their TIB, resulting in 50% of the group increasing their sleep duration. The SE group's change from baseline in sleep duration (hours:minutes) ranged from 00:52 to 01:28. Three participants in the SE group achieved a weekly average sleep duration within the 7- to 9-h recommendation. There were no significant differences in any measures of sleep duration within the control group at endpoint.

**TABLE 2:** Effects of a sleep extension intervention on outcomes of sleep duration and quality in the sleep extension group compared with the control group^1^

	Control group (*n* = 21)		Sleep extension group (*n* = 21)			
	Baseline	Change from baseline	Baseline	Change from baseline	Mean difference	*P* ^2^
Mean 7-d actigraphy						
Time in bed, h:mm	7:09 (6:51, 7:28)	−0:02 (−0:19, 0:16)	7:03 (6:45, 7:21)	0:55 (0:37, 1:12)^3^	0:56 (0:31, 1:21)	<0.001
Sleep period, h:mm	6:54 (6:36, 7:12)	0:05 (−0:23, 0:13)	6:50 (6:33, 7:08)	0:47 (0:29, 1:05)^3^	0:52 (0:27, 1:17)	<0.001
Sleep duration, h:mm	5:53 (5:36, 6:11)	−0:11 (−0:26, 0:04)	5:28 (5:48, 6:19)	0:21 (0:06, 0:36)	0:32 (0:11, 0:54)	0.004
Sleep latency,^4^ min	7.6 (5.3, 10.8)	1.8 (−1.5, 5.1)	7.7 (5.6, 9.9)	5.7 (2.3, 9.0)	3.9 (−0.9, 8.6)	0.108
Sleep duration,^4^ %	85.0 (82.5, 87.6)^5^	−0.5 (−2.4, 1.4)	88.8 (87.3, 90.4)	−4.3 (−6.2, −2.4)^3^	−3.7 (−6.5, -0.9)	0.010
Sleep efficiency,^4^ %	82.0 (79.2, 84.8)^5^	−1.3 (−3.1, 0.6)	86.2 (84.6, 87.8)	−5.4 (−7.3, −3.6)^3^	−4.2 (−6.9, −1.5)	0.003
SFI^3^	27.0 (22.8, 32.0)^5^	−0.9 (−3.1, 1.4)	19.9 (18.1, 21.9)	4.0 (1.8, 6.3)^3^	4.9 (1.6, 8.3)	0.005
Sleep questionnaires						
PSQI	6.8 (5.2, 8.3)	0.3 (−0.5, 1.0)	5.2 (4.2, 6.3)	−1.1 (−1.8, −0.4)^3^	−1.3 (−2.3, −0.3)	0.013
SHI	20.9 (18.3, 23.5)	1.0 (−1.3, 3.2)	19 (16.3, 21.7)	−3.9 (−6.2, −1.6)^3^	−4.9 (−8.1, −1.6)	0.004

1 Values are means (95% CIs). Values presented at endpoint are estimated marginal means, adjusted for baseline. “Time in bed” indicates time from “lights out” to “got up” as indicated by participants by using an event marker button on the actigraphy device; “Sleep period” indicates time from “fell asleep” to “woke up”; “Sleep duration” indicates time spent asleep within sleep period, excluding wake time; “Sleep latency” indicates time from “lights out” to “fell asleep”; “Sleep duration” (%) indicates the proportion of time spent asleep in the sleep period; “Sleep efficiency” (%) indicates the proportion of time spent asleep of time in bed. h:mm, hours:minutes; PSQI, Pittsburgh Sleep Quality Index; SFI, Sleep Fragmentation Index; SHI, Sleep Hygiene Index.

2 Differences between groups in the change from baseline were tested by ANCOVA, with baseline measurements as a covariate.

3 Significant difference within the group; baseline and endpoint data were tested by paired-samples *t* test (*P* < 0.05).

4 Values are geometric means (95% CIs). Data were log-transformed before analysis.

5 Different from the sleep extension group at baseline, as assessed by independent-samples *t* test (*P* < 0.05).

#### Sleep quality

The changes from baseline for measures of sleep quality, including sleep duration (percentage), sleep efficiency, and SFI, indicate poorer sleep quality in the SE group than in the control group. However, there was no significant difference in sleep latency between groups as a result of the intervention. Within-group analysis of the control group showed no significant changes in sleep quality at endpoint.

#### Subjective measures of sleep and sleep hygiene

The change from baseline in the SE group's Pittsburgh Sleep Quality Index and Sleep Hygiene Index scores was significantly lower in the SE group in comparison to the control group. There were no within-group differences in the control group.

### Dietary intake

The effects of the SE intervention on dietary intake are presented in **Table 3**. The reduction in the reported intake of free sugars (grams) was significantly different from control (*P* = 0.042, Cohen's *d* = 0.79). Within-group comparisons showed a significant decrease in reported free-sugars intake (−9.6 g; 95% CI: −16.0, −3.1 h; *P* = 0.002) in the SE group at the end of the intervention period compared with baseline. The change from baseline for the reported intake of protein (percentage of energy) was significantly lower in the control group compared with the SE group (*P* = 0.018, Cohen's *d* = 0.92); however, there was no difference between groups in grams of protein consumed. There were trends for a reduced intake of carbohydrates (grams; *P* = 0.083) and fat (percentage of energy; *P* = 0.074) in the SE group in comparison to the control group.

**TABLE 3:** Effects of a sleep extension intervention on dietary intake and quality in the sleep extension group compared with the control group^1^

	Control group (*n* = 21)		Sleep extension group (*n* = 21)				
	Baseline	Change from baseline	Baseline	Change from baseline	Mean difference^2^	*P* ^2^	*P* ^3^
Energy, kcal/d	1743 (1556, 1929)	−6.8 (−201.7, 188.0)	1846 (1645, 2047)	−176.4 (−371.2, 18.4)	−169.6 (−469.1, 130.0)	0.259	0.156
Protein							
g/d^4^	68.8 (60.0, 78.9)	−6.1 (−16.5, 4.3)	76.6 (65.8, 89.1)	−1.5 (−11.9, 8.9)	4.5 (11.5, 20.6)	0.570	0.616
% of energy	16.6 (14.8, 18.4)	−1.9 (−3.7, −0.03)	17.7 (15.6, 19.7)	1.6 (−0.3, 3.4)	3.4 (0.6, 6.2)	0.018	0.027
Carbohydrates							
g/d	195.5 (178.3, 212.7)	3.1 (−15.3, 21.4)	212.0 (188.0, 236.1)	−22.0 (−40.3, −3.6)	−25.0 (−53.4, 3.4)	0.083	0.041
% of energy	45.6 (43.0, 48.2)	0.4 (−1.7, 2.6)	46.3 (43.8, 48.8)	0.2 (−1.9, 2.3)	−0.2 (−3.5, 3.1)	0.898	0.832
Sugar							
g/d^4^	71.8 (62.4, 82.7)	−2.8 (−13.4, 7.8)	74.0 (62.2, 88.1)	−14.3 (−24.9, −3.6)	−11.5 (−27.8, 4.9)	0.164	0.072
% of energy	15.8 (13.7, 18.0)	−1.1 (−3.2, 1.1)	16.6 (14.1, 19.2)	−0.8 (−2.9, 1.4)	0.3 (−3.0, 3.5)	0.867	0.764
Free sugars							
g/d^4^	25.9 (16.0, 31.9)	0.7 (−5.7, 7.2)	25.3 (17.0, 38.2)	−9.6 (−16.0, −3.1)^5^	−10.3 (−20.2, −0.4)	0.042	0.031
% of energy	6.2 (4.9, 7.5)	−0.1 (−1.5, 1.3)	6.3 (5.2, 7.4)	−1.5 (−2.9, −0.1)	−1.4 (−3.5, 0.7)	0.181	0.153
Fiber,^4^ g/d	18.2 (15.4, 21.5)	−1.3 (−4.3, 1.7)	20.7 (17.6, 24.4)	−3.6 (−6.6, −0.6)	−2.3 (−6.9, 2.4)	0.329	0.154
Fat							
g/d^4^	66.0 (56.1, 77.8)	1.6 (−9.7, 13.0)	67.7 (57.4, 79.9)	−10.7 (−22.0, 0.7)	−12.3 (−29.8, 5.2)	0.162	0.079
% of energy	35.5 (32.8, 38.3)	1.0 (−1.2, 3.1)	34.6 (32.1, 37.2)	−2.1 (−4.2, 0.1)	−3.0 (−6.4, 0.3)	0.074	0.037
Saturated fat							
g/d	25.1 (20.4, 29.8)	−0.2 (−4.6, 4.1)	23.7 (20.3, 27.2)	3.1 (−7.4, 1.2)	−2.9 (−9.5, 3.8)	0.390	0.272
% of energy	12.7 (11.1, 14.3)	0.2 (−1.2, 1.6)	11.5 (10.4, 12.5)	−0.7 (−2.1, 0.7)	−0.9 (−3.1, 1.3)	0.421	0.407
Alcohol^6^							
g/d	10.7 (0.8, 22.4)	−2.7 (−6.2, 0.8)	6.2 (0.1, 15.4)	0.6 (−2.9, 4.1)	3.3 (−2.1, 8.8)	0.226	0.282
% of energy	3.7 (0.3, 7.8)	−0.5 (−2.0, 1.0)	1.9 (0.02, 6.1)	0.4 (−1.1, 2.0)	0.9 (−1.4, 3.3)	0.432	0.434
Caffeine,^6^ mg/d	93.1 (20.7, 143.7)	−14.6 (−32.8, 3.6)	92.5 (20.0, 120.3)	−9.4 (−27.6, 8.8)	5.2 (−22.8, 33.2)	0.709	0.875
Eating Choices Index^6^	14.5 (12.0, 17.8)	−0.3 (−1.4, 0.9)	14.5 (12.0, 16.0)	−0.1 (−1.2, 1.0)	0.1 (−1.7, 1.9)	0.895	
UK guidelines	19.6 (17.0, 22.1)	−2.5 (−4.8, −0.2)	19.7 (17.4, 22.0)	1.3 (−1.0, 3.6)	3.8 (0.3, 7.4)	0.036	
adherence score							

1 Values are means (95% CIs). There were no significant differences between groups at baseline.

2 Differences in the change from baseline between groups were tested by ANCOVA, with baseline measurements and change in time in bed as covariates. The mean difference represents sleep extension change from baseline minus control change from baseline.

3 Plausible reporters [*n* = 18 controls (86%), *n* = 20 sleep extension participants (95%)].

4 Baseline values are geometric means (95% CIs). Data were log-transformed.

5 Significant difference within the group; baseline and endpoint data were tested by paired-samples *t* test (*P* < 0.05).

6 Baseline data were nonparametric; values presented are unadjusted medians (upper, lower quartiles).

Four implausible reporters were identified (*n* = 3 in the control group, *n* = 1 in the intervention group). A sensitivity analysis of plausible reporters showed a significantly lower change from baseline in reported intakes of free sugars (mean difference: −11.8 g; 95% CI: −22.4, −1.1 g; *P* = 0.031, Cohen's *d* = 0.89), carbohydrate (mean difference: −28.5 g; 95% CI: −55.8, −1.2 g; *P* = 0.041, Cohen's *d* = 0.40), and fat (percentage of energy; mean difference: −3.7% of energy; 95% CI: −7.2%, −0.2% of energy; *P* = 0.037, Cohen's *d* = 0.89).

### Diet quality and UK dietary guidelines adherence

The change from baseline for the UK dietary guidelines adherence score was significantly higher in the SE group in comparison to the control group (*P* = 0.036, Cohen's *d* = 0.80) at endpoint (Table 3). The score increase at endpoint was due to the change from baseline in the free-sugars component of the score in the SE group (3.3; 95% CI: 2.6, 4.0) compared with the control group (−0.5; 95% CI: −1.3, 0.4) (*P* = 0.028, Cohen's *d* = 0.88). There was no significant difference in Eating Choices Index scores between the control and intervention groups.

### Anthropometric measures, energy expenditure, and physical activity

The effects of the SE intervention on anthropometric measures, energy expenditure, and physical activity are outlined in **Table 4**. We found no significant differences at endpoint between groups in weight, body composition, and waist circumference as a result of the intervention. There were also no differences at endpoint between groups’ RMR, activity, and TEE and physical activity intensity (Table 4).

**TABLE 4:** Effects of a sleep extension intervention on anthropometric measures, energy expenditure, and physical activity intensity in the sleep extension group compared with the control group^1^

	Control group (*n* = 21)		Sleep extension group (*n* = 21)		
	Baseline	Change from baseline	Baseline	Change from baseline	Mean difference
Anthropometric measures					
BMI, kg/m^2^	22.3 (21.1, 23.5)	−0.004 (−0.2, 0.2)	22.6 (21.4, 23.9)	0.1 (−0.04, 0.3)	0.1 (−0.1, 0.4)
Body weight, kg	64.1 (59.3, 69.0)	0.004 (−0.5, 0.5)	62.8 (57.5, 68.1)	0.3 (−0.1, 0.8)	0.3 (−0.4, 1.1)
Fat-free mass, kg	48.3 (43.8, 52.4)	0.05 (−0.7, 0.8)	46.9 (42.8, 51.0)	0.2 (−0.5, 0.9)	0.2 (−0.9, 1.3)
BF, %	24.1 (20.3, 28.0)	−0.1 (−1.1, 0.8)	24.8 (22.1, 27.5)	0.03 (−0.9, 0.9)	0.2 (−1.2, 1.6)
WC,^2^ cm	77.4 (73.6, 81.3)	−0.2 (−1.7, 1.3)	77.4 (73.6, 81.3)	−0.2 (−1.7, 1.3)	0.1 (−2.2, 2.3)
Energy expenditure					
RMR,^2^ kcal	1300 (1189, 1421)	5 (−48, 58)	1240 (1151, 1335)	28 (−25, 80)	23 (−58, 104)
AEE,^2^ kcal	707 (572, 875)	−27 (−155, 102)	664 (650, 816)	2 (−127, 131)	28 (−170, 227)
TEE,^3^ kcal	2457 (2057, 3679)	−5 (−151, 140)	2167 (1986, 2945)	7 (−138, 153)	12 (−212, 236)
PAL,^2^ TEE:RMR	1.7 (1.6, 1.8)	0.004 (−0.1, 0.1)	1.7 (1.6, 1.8)	−0.01 (−0.1, 0.1)	−0.01 (−0.1, 0.1)
Physical activity intensity, % of active time					
Vigorous^2^	0.3 (0.1, 0.8)	0.9 (−0.4, 2.0)	0.3 (0.1, 0.6)	0.5 (−0.7, 1.7)	−0.3 (−2.1, 1.5)
Moderate^2^	5.9 (4.3, 8.2)	2.2 (−0.4, 4.9)	4.1 (3.0, 5.6)	3.0 (0.3, 5.7)	0.8 (−3.4, 4.9)
Low	61.7 (58.8, 64.7)	−0.3 (−2.1, 2.7)	60.4 (57.5, 63.4)	0.2 (−2.3, 2.6)	−0.2 (−3.9, 3.5)
Sedentary	29.1 (26.1, 32.1)	−3.8 (−6.9, −0.7)	33.0 (29.4, 37.0)	−3.3 (−6.3, −0.2)	0.5 (−4.3, 5.3)

1 Values are means (95% CIs). There were no significant differences between groups at baseline. Differences in the change from baseline between groups were tested by ANCOVA, with baseline measurements and change in time in bed as covariates. There were no significant differences between groups (*P* values not shown). AEE, activity energy expenditure; BF, body fat; PAL, physical activity level; RMR, resting metabolic rate; TEE, total energy expenditure; WC, waist circumference.

2 Baseline values are geometric means. Data were log-transformed.

3 Baseline data were nonparametric. Values presented at baseline are unadjusted medians (upper, lower quartiles).

### Cardiometabolic risk, appetite hormones, and heart rate variability

There were no significant differences between groups in indicators of cardiometabolic risk or appetite hormones, as shown in **Supplemental Table 1**. Measures of HRV are outlined in **Supplemental Table 2**, and we found no significant differences between the SE and control groups.

## DISCUSSION

This SE RCT had good rates of participation, low rates of attrition, and satisfactory compliance in healthy, free-living adult short sleepers. These results show that a personalized behavioral consultation targeting sleep hygiene is a feasible lifestyle intervention and can be used to test the health effects of SE. Our pilot investigation of secondary outcomes indicates that adherence to advice to extend sleep may reduce free-sugars intake. No effects were observed on indexes of body composition, energy balance, cardiometabolic risk, appetite hormones, or heart rate variability as a result of the intervention.

To our knowledge, this is the first long-term RCT to show the feasibility of SE by addressing sleep hygiene under free-living conditions in healthy adults who are habitually short sleepers and uses objective measures of sleep to measure compliance. Previous long-term (≥1 mo) SE trials were conducted in prehypertensive adults (26, 27) or in overweight adults as part of a weight-loss program (60), sometimes relying on self-reported measures of sleep (26, 60). Previous SE interventional studies in healthy, free-living short sleepers were nonrandomized (61, 62). In the present study, the intervention increased TIB in the SE group, allowing for a longer sleep period, thus increasing sleep duration in comparison to the control group; however, they did not achieve the recommended 7–9 h of sleep. We also found that objective measures of sleep quality were modestly lower in the SE group than in the control group. The decline in sleep quality may be due to a period of adjustment to the prescribed longer TIB. This apparently negative consequence of earlier bedtimes may dissipate over time. A study conducted in young adults for 3 wk suggested that their graduated SE protocol preserved participants’ sleep quality (63). Future SE trials should investigate maintenance of or improvement in sleep quality, as well as the potential benefits of introducing gradual behavioral changes.

SE resulted in a reduction of ∼10 g of reported intakes of free sugars, equating to approximately one-third of the UK dietary guidelines’ daily allowance (64). The SE group's reduction in free-sugars intake was also the main contributing factor to the increased score for adherence to the UK dietary guidelines. However, the change in the percentage of energy from free sugars was not different between groups, suggesting that the reduction in sugary foods was not independent of reported total energy consumed. Although there were no significance differences between groups in the change in energy intake, it is possible that participating in the sleep intervention had driven changes in the SE group's dietary reporting, which may result in artefacts. The longest partial sleep deprivation RCT to date reported that a 2-wk intervention induced the consumption of excess calories from energy-dense, high-carbohydrate snacks, with no significant effect on total energy intake (65), lending support to our findings. This suggests that the observational links between short sleep and poorer-quality diets (66, 67) and increased intake of sugar (12, 68) may be causal. In addition to the observed effects on reported intakes of free sugars, there was a trend for a reduction in reported fat intake in the plausible reporters, which became significant when analyzed as a percentage of total energy. SE may thus lead to a tendency to select foods with lower fat and higher protein contents. Previous RCTs have shown enhanced brain neuronal activation in response to unhealthy food images in a sleep-deprived condition compared with a normal sleep condition (69, 70), suggesting that sleep influences reward-driven eating behavior. SE may dampen hedonic signals that drive food intake. Leptin and ghrelin have been implicated in sleep-deprivation studies but were unaffected by SE in the present study. Because we observed a decline in sleep quality in the SE group, it is possible that the extended TIB, independent of sleep quality, could limit opportunities to eat, regardless of levels of hunger. However, our interpretation of leptin and ghrelin is limited due to possible changes in circadian oscillations as a result of SE, because only single fasting morning samples were taken per participant, albeit collected at similar times. Our findings need to be interpreted with caution, because participating in an intervention at a nutrition department may have altered participants’ tendency to bias their dietary reports to a healthier diet.

We found no significant differences in markers of energy balance between groups, including body weight and composition, energy expenditure, and physical activity levels. Experimental sleep-restriction studies reported inconsistent findings, with some suggesting decreased physical activity (71, 72) in those who are sleep deprived and others reporting no changes in energy expenditure when assessed by using doubly labeled water (65, 73). Our study design precludes quantifying net energy balance, because our assessment of the mean 2-d energy expenditure may not reflect the mean 7-d reported dietary intake because humans are in constant energy flux (74). Although we found no difference in weight over a 1-mo period, diet quality may influence an individual's ability to maintain energy balance over the longer term (75).

There are several limitations to the present study. First, changes in behavior variables may have been due to the “Hawthorne effect,” as previously reported by Cizza et al. (76). In addition, the lack of ability to blind participants to the allocation of the SE intervention may have introduced confounding effects in the control condition by indirectly stimulating an interest in sleep. To minimize this possible effect, terms such as “sleep hygiene” were avoided during interactions with the control group. Future SE trials may consider blinding assessors to minimize sources of bias. In addition, the sample was predominantly white, young women recruited from a university setting, and this may limit generalizability to other sociodemographic groups. There are noteworthy limitations to the use of dietary records, because these are prone to reporting bias. Although we identified plausible reporters, their reported intakes are not necessarily accurate. Moreover, a 1-mo intervention may have been inadequate to allow for adaptation, warranting longer future trials. Finally, secondary outcomes were considered pilot investigations and we may have been underpowered to detect differences between groups.

To our knowledge, this is the only RCT to date to investigate SE by using a personalized behavioral approach in healthy adult short sleepers. It was conducted under free-living conditions and presents ecologically valid evidence. Further strengths include the objective measurement of sleep, physical activity, and energy expenditure (RMR and TEE). Moreover, we generated SDs for a range of outcomes to be used in power calculations for future RCTs.

We conclude that a tailored behavioral SE intervention targeting sleep hygiene is feasible in healthy, free-living young adults, which shows the utility of including sleep hygiene guidelines in public health messages. We also showed that SE may reduce reported intake of free sugars, consequently improving diet quality and supporting the theory that diet may be a key mediator in the relation between short sleep and metabolic disease. The results of this trial need to be confirmed by using methods less prone to bias, necessitating inpatient dietary assessment or biomarker studies. A secondary aim of this study was to explore mechanisms linking SE and components of energy balance and dietary intake. This trial did not aim to determine the long-term impact of sleeping habits on weight change. Larger and longer-term RCTs are needed to examine the effects of continued adherence to sleep hygiene advice and preservation of sleep quality on energy balance, particularly in “at risk” populations.

## Supplementary Material

Supplemental data

## Notes

Supported by funding from the British Nutrition Foundation.

Supplemental Tables 1 and 2 are available from the “Supplementary data” link in the online posting of the article and from the same link in the online table of contents at https://academic.oup.com/ajcn/.

**Abbreviations used**
KCL: King's College London
RCT: randomized controlled trial
RMR: resting metabolic rate
SE: sleep extension
SFI: Sleep Fragmentation Index
TEE: total energy expenditure
TIB: time in bed
