# Supplementary material for: Sleep extension is a feasible lifestyle intervention in free-living adults who are habitually short sleepers: a potential strategy for decreasing intake of free sugars? A randomized controlled pilot study
Source: Am J Clin Nutr. 2018 Jan 10;107(1):43–53. doi: 10.1093/ajcn/nqx030 (PMC5972593; doi:10.1093/ajcn/nqx030)
Supplement: Supplemental data [file nqx030_supp.docx]

Online Supporting Material

**Supplemental Table 1.** Indicators of cardio-metabolic risk and appetite hormones in the control and sleep extension groups.

|  | Control n = 21 | | Sleep Extension n = 21 | | Mean  Difference |
| --- | --- | --- | --- | --- | --- |
|  | Baseline | Change From Baseline | Baseline | Change From Baseline |  |
| Blood Pressure |  |  |  |  |  |
| SBP (mmHg) ^1^ | 110.3 (104.7, 116.1) | -4.2 (-7.5, -1.0) | 112.9 (106.7, 119.4) | -2.3 (-5.7, 1.1) | 1.9 (-3.2, 7.1) |
| DBP (mmHg) ^1^ | 67.4 (62.7, 72.5) | -1.6 (-4.3, 1.1) | 69.2 (65.3, 73.4) | -0.5 (3.2, 2.3) | 1.1 (-3.0, 5.3) |
| Blood Measures ^2^ |  |  |  |  |  |
| Glucose (mmol/L) | 5.0 (4.7, 5.3) | 0.1 (-0.1, 0.3) | 5.0 (4.8, 5.2) | 0.1 (-0.1, 0.3) | 0.02 (-0.3, 0.3) |
| NEFA (mmol/L) ^3^ | 0.3 (0.1, 0.4) | 0.1 (-0.1, 0.2) | 0.4 (0.2, 0.5) | 0.004 (-0.1, 0.1) | -0.1 (-0.3, 0.1) |
| TC (mmol/L) ^1^ | 4.8 (4.4, 5.3) | -0.1 (-0.4, 0.2) | 4.6 (4.2, 5.0) | 0.1 (-0.2, 0.4) | -0.1 (-0.3, 0.6) |
| TAG (mmol/L) | 0.8 (0.6, 0.9) | -0.1 (-0.2, 0.1) | 0.8 (0.7, 0.9) | 0.1 (-0.1, 0.2) | 0.2 (-0.1, 0.4) |
| HDL-C (mmol/L) | 1.8 (1.6, 1.9) | -0.1 (-0.1, 0.04) | 1.7 (1.5, 1.9) | 0.03 (-0.1, 0.1) | 0.1 (-0.1, 0.2) |
| LDL-C (mmol/L) | 2.7 (2.2, 3.2) | 0.01 (-0.2, 0.3) | 2.6 (2.2, 3.0) | 0.3 (-0.2, 0.3) | 0.01 (-0.4, 0.4) |
| TC:HDL-C Ratio ^1^ | 2.7 (2.4, 3.1) | 0.1 (-0.1, 0.2) | 2.8 (2.5, 3.0) | 0.00 (-0.2, 0.2) | -0.1 (-0.3, 0.2) |
| Insulin (mIU/L) ^1^ | 8.4 (6.3, 11.3) | -1.9 (-4.1, 0.2) | 9.0 (7.3, 11.2) | 0.7 (-1.4, 2.7) | 2.6 (-0.8, 6.0) |
| HOMA-IR ^3^ | 1.8 (1.1, 2.9) | -0.4 (-0.9, 0.2) | 2.1 (1.3, 2.8) | 0.2 (-0.3, 0.7) | 0.5 (-0.3, 1.4) |
| Cortisol (nmol/L) ^3^ | 300 (231, 472) | -47 (-124, 30) | 261 (182, 417) | -18 (-92, 56) | 29 (-91, 149) |
| C-peptide (mIU/L) ^1^ | 392 (329, 467) | -36 (-90, 18) | 376 (319, 444) | 19 (-33, 71) | 55 (-30, 140) |
| Leptin (µg/L) ^1^ | 0.9 (0.6, 1.3) | -0.3 (-0.5, -0.03) | 0.9 (0.8, 1.2) | 0.04 (-0.2, 0.3) | 0.3 (-0.1, 0.7) |
| Ghrelin (ng/L) ^1^ | 1767 (1441, 2167) | 203 (4.6, 401) | 1880 (1569, 2253) | -41 (-225, 143) | -244 (-548, 61) |
| Digital Volume Pulse |  |  |  |  |  |
| Stiffness Index (m/sec) | 6.5 (5.8, 7.2) | -0.2 (-0.6, 0.3) | 6.0 (5.5, 6.5) | -0.2 (-0.6, 0.2) | -0.03 (-0.7, 0.6) |
| Reflection Index (%) | 74.3 (69.3, 79.3) | 2.4 (-5.2, 10.0) | 70.1 (63.8, 76.5) | -1.4 (-8.8, 6.0) | -3.8 (-15.4, 7.8) |

*Abbreviations:* SBP, systolic blood pressure; DBP, diastolic blood pressure; NEFA, non-esterified fatty acids; TC, total cholesterol; TAG, triglycerides; HDL-C, high density lipoprotein cholesterol; LDL-C, low density lipoprotein cholesterol; TC:HDL, total cholesterol to high density lipoprotein cholesterol ratio; HOMA-IR, homeostatic model assessment of insulin resistance.

Values are reported as mean (95% CI). There were no significant differences between groups at baseline. or as a result of the intervention.
Difference in the change from baseline between was tested by ANCOVA, with baseline measurements and change in Time in Bed as covariates. There were no significant differences between groups (*P* values not shown).
^1^ Baseline values are geometric means; data were log transformed.
^2^ Data from n = 8 participants absent from analysis of control and intervention groups (Control: n = 16, Intervention: n = 18) due to inability to draw blood.
^3^ Baseline data were non-parametric; Values presented are unadjusted medians (upper, lower quartiles).

Online Supporting Material

**Supplemental Table 2.** Heart rate variability in the control and sleep extension groups.

|  | Control  n = 18 | | Sleep Extension  n = 19 | | Mean Difference |
| --- | --- | --- | --- | --- | --- |
|  | Baseline | Change From Baseline | Baseline | Change From Baseline |  |
| **Sleep time** |  |  |  |  |  |
| IBI (ms) ^1^ | 960.9  (909.6, 1015.0) | 9.9  (-44.2, 64.0) | 957.0  (882.0, 1038.4) | -10.5  (-62.9, 41.9) | -20.4  (-101.1, 60.3) |
| *Beat-to-beat variability* |  |  |  |  |  |
| PNN50 (%) ^2^ | 28.5 (15.0, 46.6) | 4.4 (-4.0, 12.9) | 32.4 (12.5, 42.6) | -3.4 (-11.6, 4.8) | -7.9 (-20.5, 4.8) |
| RMSSD (ms) ^1^ | 52.5 (44.5, 61.8) | 5.3 (-4.8, 15.5) | 52.5 (44.1, 62.6) | -4.5 (-14.3, 5.4) | -9.8 (-25.0, 5.4) |
| HF (ms^2^) ^1^ | 971.1  (715.8, 1317.6) | 282.9  (-197.6, 763.5) | 876.4  (591.3, 1299.2) | -134.6  (-600.5, 331.3) | -417.5  (-1134.6, 299.5) |
| *Longer phase variability* |  |  |  |  |  |
| SDANN (ms) ^1^ | 46.8 (38.5, 57.0) | 9.2 (-0.1, 18.5) | 39.0 (29.1, 52.3) | 2.2 (-6.8, 11.2) | -7.0 (-21.1, 7.1) |
| LF (ms^2^) ^2^ | 893.0  (701.1, 1671.8) | 248.6  (-73.2, 570.5) | 807.6  (505.8, 1529.5) | -107.0  (-418.9, 204.9) | -355.6  (-126.8, 838.0) |
| *Symp/parasym balance* |  |  |  |  |  |
| Log (LF:HF) | 0.01 (-0.07, 0.1) | -0.021 (-0.1, 0.1) | -0.04 (-0.1, 0.1) | 0.006 (-0.1, 0.1) | 0.03 (-0.2, 0.2) |
| **Day time** |  |  |  |  |  |
| IBI (ms) ^1^ | 784.6  (729.3, 844.1) | -14.2  (-62.1, 33.7) | 750.7  (696.2, 809.4) | -22.5  (-68.9, 23.9) | -8.3  (-80.7, 64.1) |
| *Beat-to-beat variability* |  |  |  |  |  |
| PNN50 (%) ^1^ | 17.2 (11.6, 25.5) | -4.7 (-10.4, 2.1) | 11.5 (7.0, 19.0) | -2.6 (-8.6, 3.5) | 1.6 (-7.9, 11.0) |
| RMSSD (ms) ^2^ | 49.2 (38.5, 60.2) | -5.2 (-12.5, 2.0) | 47.2 (29.1, 60.0) | -2.4 (-9.4, 4.6) | 2.8 (-8.2, 13.8) |
| HF (ms^2^) ^1^ | 704.7  (500.5, 992.2) | -149.1  (-390.3, 92.2) | 523.6  (339.3, 808.1) | -194.3  (-428.1, 39.5) | -45.2  (-406.5, 316.1) |
| *Longer phase variability* |  |  |  |  |  |
| SDANN (ms) ^1^ | 62.7 (52.2, 75.4) | -4.2 (-17.8, 9.5) | 59.6 (48.8, 72.8) | -6.6 (-19.8, 6.7) | -2.4 (-22.9, 18.1) |
| LF (ms^2^) ^2^ | 1081.7  (725.8, 1672.6) | 33.5  (-281.5, 348.5) | 1078.9  (522.4, 1877.0) | -100.8  (-406.2, 204.6) | -134.3  (-603.9, 335.2) |
| *Symp/parasym balance* |  |  |  |  |  |
| Log (LF:HF) | 0.18 (0.1, 0.3) | 0.1 (-0.03, 0.2) | 0.3 (0.2, 0.4) | 0.03 (-0.1, 0.1) | -0.1 (-0.2, 0.1) |

*Abbreviations:* IBI, interbeat interval, also known as R interval, the time interval between R spikes of the QRS complex of the electrocardiogram; ms, HR, heart rate; bpm, beats per minute; NN, normal-to-normal (similar to RR but on normalised IBI data); SDNN, standard deviation of all NN intervals; RMSSD, square root of the mean of the sum of squares with differences between adjacent NN intervals; PNN50, percentage of adjacent NN intervals that differed by >50 ms; LF, low-frequency power; HF, high frequency power; symp-parasymp balance; sympathetic to parasympathetic balance.

Values are reported as mean (95% CI). There were no significant differences between groups at baseline.
Difference in the change from baseline between was tested by ANCOVA, with baseline measurements and change in Time in Bed as covariates. There were no significant differences between groups (*P* values not shown).
^1^ Baseline values are geometric means; data were log transformed prior to analysis.
^2^ Baseline data were non-parametric; Values presented are unadjusted medians (upper, lower quartiles).
Data from n = 5 participants (n = 3 control, n = 2 sleep extension) absent from analysis due to high level of noise in IBI recording.
